# Effect of topographic comparison of electroencephalographic microstates on the diagnosis and prognosis prediction of patients with prolonged disorders of consciousness

**DOI:** 10.1111/cns.14421

**Published:** 2023-09-07

**Authors:** Yi Ling, Xinrui Wen, Jianghui Tang, Zhengde Tao, Liping Sun, Hailiang Xin, Benyan Luo

**Affiliations:** ^1^ Department of Neurology, First Affiliated Hospital, School of Medicine Zhejiang University Hangzhou China; ^2^ Zhejiang Provincial Key Laboratory of Pancreatic Disease Zhejiang University School of Medicine First Affiliated Hospital Hangzhou China; ^3^ Department of Neurology First People's Hospital of Wenling Zhejiang China; ^4^ Department of Rehabilitation Hangzhou Mingzhou Brain Rehabilitation Hospital Hangzhou China; ^5^ The MOE Frontier Science Center for Brain Science and Brain‐Machine Integration Zhejiang University Hangzhou China

**Keywords:** diagnosis, EEG, microstates, prognosis, prolonged disorders of consciousness, topographic difference

## Abstract

**Aims:**

The electroencephalography (EEG) microstates are indicative of fundamental information processing mechanisms, which are severely damaged in patients with prolonged disorders of consciousness (pDoC). We aimed to improve the topographic analysis of EEG microstates and explore indicators available for diagnosis and prognosis prediction of patients with pDoC, which were still lacking.

**Methods:**

We conducted EEG recordings on 59 patients with pDoC and 32 healthy controls. We refined the microstate method to accurately estimate topographical differences, and then classify and forecast the prognosis of patients with pDoC. An independent dataset was used to validate the conclusion.

**Results:**

Through optimized topographic analysis, the global explained variance (GEV) of microstate E increased significantly in groups with reduced levels of consciousness. However, its ability to classify the VS/UWS group was poor. In addition, the optimized GEV of microstate E exhibited a statistically significant decrease in the good prognosis group as opposed to the group with a poor prognosis. Furthermore, the optimized GEV of microstate E strongly predicted a patient's prognosis.

**Conclusion:**

This technique harmonizes with the existing microstate analysis and exhibits precise and comprehensive differences in microstate topography between groups. Furthermore, this method has significant potential for evaluating the clinical prognosis of pDoC patients.

## Graphical abstract

We improved the microstate method for measuring global explained variance (GEV), and the optimized GEV of microstate E strongly predicted the prognosis of patients with prolonged disorders of consciousness (pDoC).

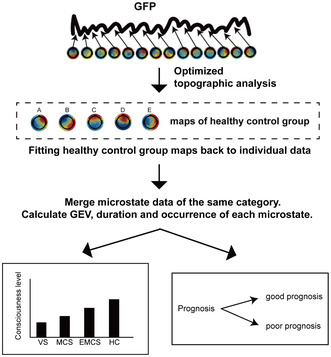

## 1 INTRODUCTION

Disorders of consciousness (DoC) arise from profound brain damage caused by traumatic brain injury, cerebrovascular disease, or cardiovascular arrest. The spectrum of disorders of consciousness (DoC) encompasses coma, unresponsive wakefulness syndrome/vegetative state (UWS/VS), and minimally conscious state (MCS).^1^ However, the misdiagnosis rates remain high at 37%–43%, and there are limited prognostic assessment tools available.^2^, ^3^, ^4^

Electroencephalographic (EEG) microstates are fundamental neural building blocks of consciousness.^5^ These stable scalp potential configurations last for 60–120 ms before transitioning to other stable topographies called metastable states, which are important for conscious experiences.^5^ Microstates are regarded as the fundamental units or “atoms of thought.” According to the hypothesis of “atoms of thought,” the activation of the network during a given microstate corresponds to distinct states of consciousness, with each microstate being linked to a specific category of mental activity that collectively comprises the conscious state.^6^ Analysis of clusters consistently reveals four configurations, called EEG microstate classes A to D.^7^, ^8^

Microstate temporal dynamics are sensitive to neuropsychiatric conditions^9^, ^10^ and altered states of consciousness.^11^, ^12^, ^13^ Schizophrenia patients and their siblings have a higher prevalence of class C and a lower prevalence of class D.^9^ All classes of Lewy body dementia have longer microstate durations in comparison with controls, but the occurrence of each class is lower than that in controls.^14^ In the case of prolonged disorders of consciousness (pDoC), there were fewer classes of microstates,^15^ and microstate D proved to be the most precise metric for discerning between VS/UWS and MCS.^16^ Thus, analyzing microstate temporal parameters provides a useful macroscale window for studying abnormal brain activity.

Although differences in the temporal parameters of EEG microstates between groups were identified by conventional microstate analysis,^5^, ^7^ a significant but often overlooked aspect is that all studies focused solely on topographic map comparisons to their group maps, ignoring the differences between groups. Thus, existing microstate analysis lacked specific and concrete indicators measuring topographic differences, limiting their application for diagnosis and prognosis prediction.

In our research, we improved the microstate method for measuring GEV, and the optimized GEV of microstate E had a significant role in predicting the outcome of patients with DoC. The optimized topographic analysis is complementary to the current microstate analysis and may serve as new tools to better classify and predict the prognosis of patients with brain diseases (e.g., pDoC patients).

## 2 METHODS AND MATERIALS

### 2.1 Participants

All patients with pDoC were enrolled at the Hangzhou Mingzhou Brain Rehabilitation Hospital, while healthy controls were recorded at the First Affiliated Hospital of Zhejiang University. The validation queue utilized in this study had been previously employed in other scholarly works to examine phase coherence under identical experimental conditions and with the same equipment.^17^ Including criteria are as follows: >28‐day enduring consciousness, stable vital signs, and intact cranium. Excluding criteria are as follows: hearing impairment, neurodevelopmental/neurological conditions and recent use of sedatives/muscle relaxants within 24 h prior to study. A follow‐up assessment using the Glasgow Outcome Scale (GOS) was conducted by telephone with the patient's families 6 months after the EEG collection.^18^ A GOS score ≥4 points is considered to indicate a good prognosis, while a GOS score <4 points is considered to indicate a poor prognosis.^19^

### 2.2 Ethical consideration

The guardians of patients and healthy participants signed informed consent forms and could quit at any time. The investigation received ethical approval from the Ethical Committee of the First Affiliated Hospital of Zhejiang University and Hangzhou Mingzhou Brain Rehabilitation Hospital (NCT05949528). All procedures were performed in compliance with the principles outlined in the Declaration of Helsinki.

### 2.3 Microstate extraction

Cartool was used to analyze EEG microstates. The primary objective of Cartool is to examine the spatial characteristics of the electric fields in the brain and to quantitatively evaluate alterations in field topographies over time, under varying experimental conditions, or across different populations.^20^

#### 2.3.1 Fitting

Agglomerate hierarchical clustering was applied to maps labeled as global field power (GFP) peaks to extract distinct templates, maximize the variance of GFP peak topographies, and reduce the number of templates by one during each iteration. Finally, our study found that a value of *k* = 5 exhibited the highest level of map reliability among participants and groups.

#### 2.3.2 Backfitting

##### Method 1: Conventional method

Aggregate microstates (*k* = 5) of the groups were placed back to primordial EEG recordings. As part of this process, EEG frames were assigned to microstate labels based on spatial correlations, time points were labeled based on the group template that they corresponded to, and a microstate sequence was generated for further analysis. Segments exceeding a duration of two timeframes (8 ms) were bifurcated into two, with each segment exhibiting the greatest spatial correlation with its adjacent segment, and were subsequently reclassified. Segments shorter than two timeframes were discarded.^21^ To solve the flaw of neglecting the difference between groups, we introduced two new methods of microstate analysis.

##### Method 2: Individual clustering method

This is a new method that measures differences in topography at an individual level. We first clustered the five maps of each patient, then we chose to use the *k* = 5 aggregate maps of healthy controls as templates and assigned each individual aggregate map (*k* = 5) to the most similar template based on the maximum absolute Pearson correlation value. This provided us with a label (microstate) sequence for each individual, based on the healthy control group.

Although the individual clustering method can be effective, it has limitations. Clustering individual topographic maps first can lead to the misclassification of certain maps due to the consideration of the maximum GEV feature. For example, a map that should be assigned to microstate A after matching with the template microstate may be classified incorrectly as microstate B due to the prioritization of the maximum GEV value. This ultimately increased the risk of type I errors. To address this, our approach has been further refined for improved reliability and accuracy.

##### Method 3: Optimized topographic analysis

Taking into consideration the limitations of the initial clustering method applied to individual maps, we decided to utilize the aggregate classes (*k* = 5) of healthy controls as templates. Subsequently, the raw EEG recordings of each participant were allocated to one of the aforementioned templates by utilizing the highest absolute Pearson correlation value. This measure ensured that every time point was appropriately labeled based on the corresponding template of healthy controls. Our refined approach not only optimized the calculation of GEV but also reduced the risk of type I errors, making it the optimal method.

When calculating GEV, the split microstates were considered as the same microstate, rather than being treated as two distinct microstates. The duration and occurrence were calculated by the EEGLAB plugin in MATLAB R2013b. To further compare the differences in computing duration and occurrence between the EEGLAB plugin and Cartool software, we additionally utilized two methods that employ the Cartool software for computing these microstate parameters.

### 2.4 Statistics

We assessed data normality distribution using the Kolmogorov–Smirnov test and tested all data for normality. Means and standard deviations were reported for normally distributed data, while medians and interquartile ranges were reported for non‐normally distributed data. A one‐way ANOVA and independent sample *t*‐test were performed on normally distributed data, while the Kruskal–Wallis test was used for non‐normally distributed data. Sex and etiology were analyzed using the chi‐squared test. Classification and prediction performances were assessed using receiver operating characteristic (ROC) curves. The Bonferroni method was applied for multiple comparisons. A significance level of *p* < 0.05 was established.

## 3 RESULTS

### 3.1 Clinical data showed no significant differences among different consciousness groups

We enrolled 63 patients with pDoC (13 in EMCS, 19 in MCS, and 31 in VS/UWS) and 32 healthy controls. Clinical data for each group were presented in Table 1. A statistically significant difference in CRS‐R scores was observed among the three groups with impaired consciousness; the EMCS group exhibited the highest scores, while the VS/UWS group exhibited the lowest scores (*p* < 0.001). The average age and the percentage of male individuals had no significant difference among HC, EMCS, MCS, and VS/UWS groups. Regarding etiology, the EMCS cohort comprised 69.2% trauma and 30.8% vascular cases, while the MCS consisted of 42.1% trauma and 57.9% vascular cases, and the VS/UWS encompassed 58.1% trauma and 41.9% vascular cases. The average duration of post‐injury and the region of cerebral impairment had no significant difference among EMCS, MCS, and VS groups.

**TABLE 1:** Baseline patient demographic and clinical data.

Characteristics	HC (*n* = 32)	EMCS (*n* = 13)	MCS (*n* = 19)	VS (*n* = 31)	*p*‐Value
Age	66.72 (4.48)	65.92 (4.03)	67.84 (4.90)	66.45 (6.41)	0.741
Sex (male %)	50	61.5	42.1	51.6	0.756
Etiology					0.293
Traumatic (%)		69.2	42.1	58.1	
Vascular (%)		30.8	57.9	41.9	
Months after injury		8.33 (1.07)	8.14 (1.44)	7.84 (1.20)	0.446
Area of brain injury					0.994
Left (%)		23.1	26.3	22.6	
Right (%)		30.8	26.3	25.8	
Combined (%)		46.2	47.4	51.6	
CRS‐R					
Total score		23 (23, 24)	13 (12, 16)	7 (6, 8)	<0.001
Auditory		4 (4, 4)	2 (2, 2)	2 (2, 2)	<0.001
Visual		5 (4, 5)	3 (2, 3)	1 (0, 1)	<0.001
Motor		6 (5, 6)	5 (2, 5)	1 (1, 2)	<0.001
Oromotor/verbal		3 (3, 3)	1 (0, 2)	0 (0, 1)	<0.001
Communication		2 (2, 2)	1 (0, 1)	0 (0,1)	<0.001
Arousal		3 (3, 3)	2 (2, 2)	1 (0,1)	<0.001

*Note*: One‐way ANOVA for age and months after injury analysis; chi‐squared for sex and etiology analysis; Kruskal–Wallis tests for CRS‐R analysis.

Abbreviations: CRS‐R, Coma Recovery Scale–Revised; EMCS, exit from minimal consciousness state; HC, healthy controls; MCS, minimally conscious state; VS, vegetative state.

### 3.2 Two topographic maps of microstate E but no microstate D were identified in the VS/UWS group through resting‐state topography estimation

**FIGURE 1:**
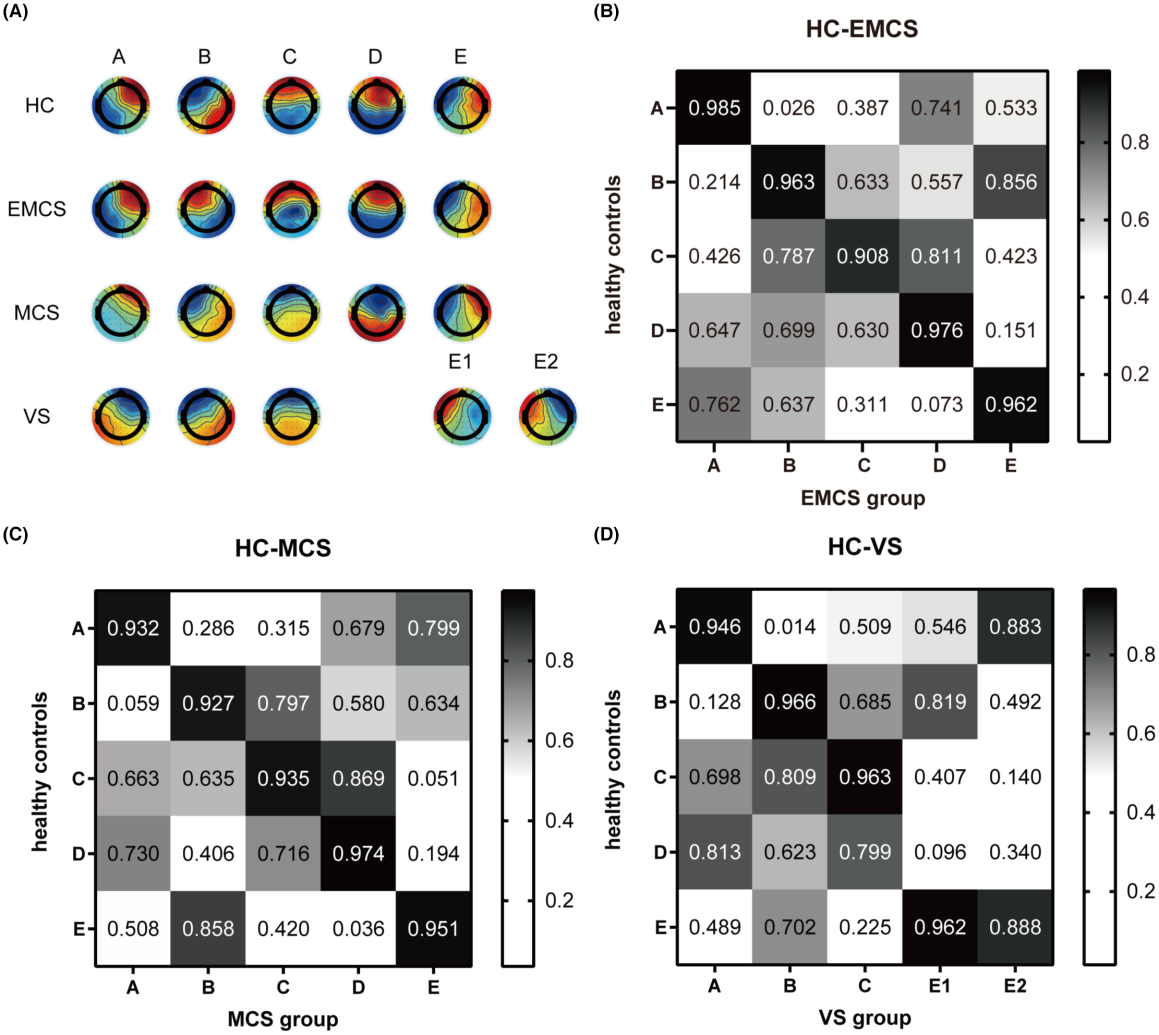
Resting‐state topography estimation. (A) Topographic maps of the five EEG microstates for groups. (B–D) Coefficients of spatial correlation between NC and EMCS (B), NC and MCS (C), and NC and VS (D). EMCS, exit from minimal consciousness state; HC, healthy controls; MCS, minimally conscious state; VS, vegetative state.

We identified five canonical microstates (Figure 1A) across groups, corresponding to conventional microstate topographies previously reported in the literature.^7^, ^8^, ^22^ Matched maps showed high similarity between each other. The five topographic maps of the EMCS and MCS groups could correspond one by one to the five topographic maps of the control group, while none of the topographies of the VS/UWS group were assigned to microstate D of the control group based on the maximal spatial correlation coefficient, but there were two topographic maps matched to microstate E (Figure 1B–D).

### 3.3 The ability to distinguish diseases based on the GEV was relatively weak

**FIGURE 2:**
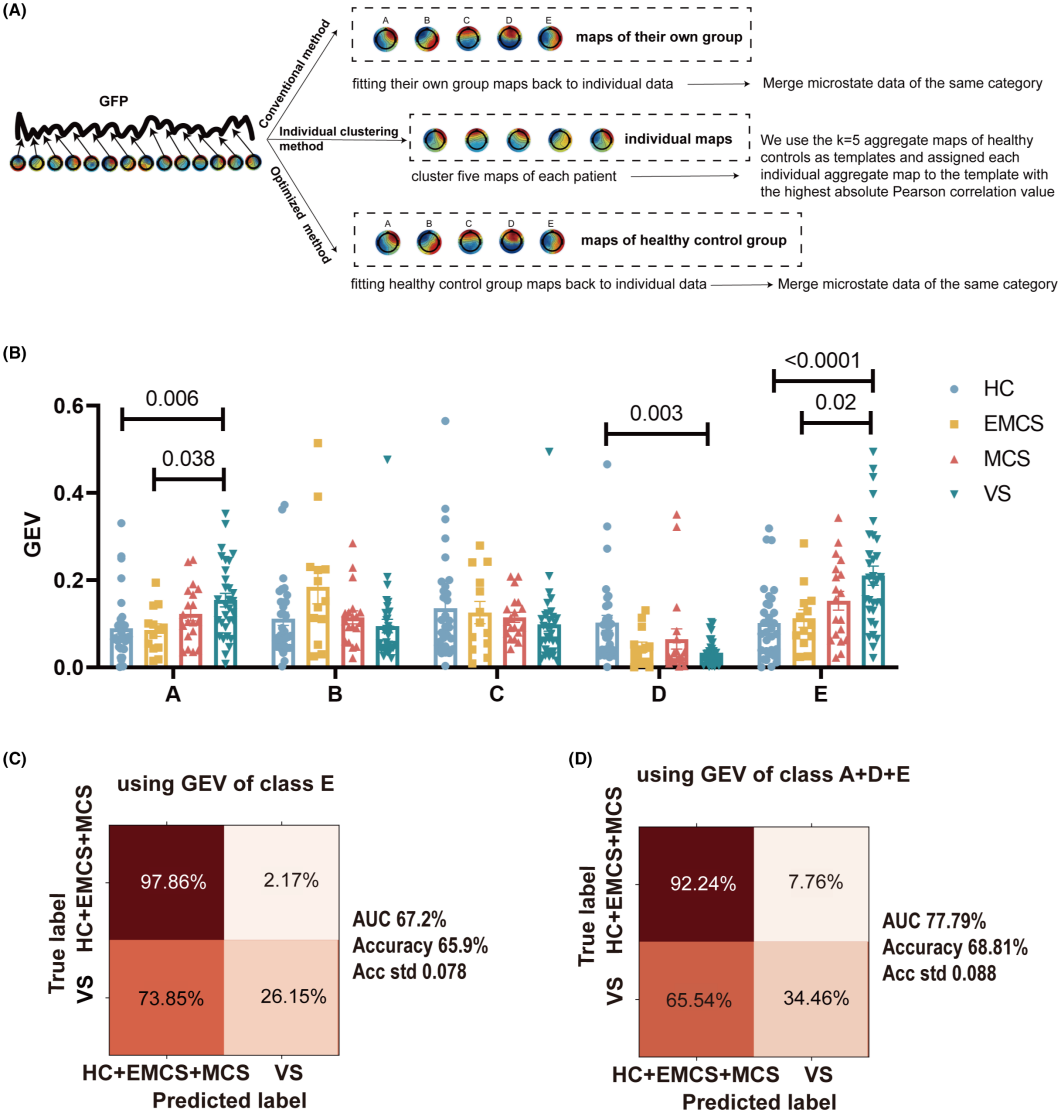
The role of optimized topographic analysis for the classification of pDoC. (A) A comparison of three methods for analyzing microstates. (B) Distribution of GEV of each microstate across groups. (C, D) GEV of microstates was used to test the classification efficiency of the SVM model for identifying VS group. Results of the confusion matrix showed 26.15%–34.46% sensitivity and 97.86%–92.24% specificity for VS diagnosis. Multiple comparisons were conducted using one‐way ANOVA in A; *p*‐values were adjusted by the Bonferroni method. AUC, the area under the curve; EMCS, exit from minimal consciousness state; GEV, global explained variance; HC, healthy controls; MCS, minimally conscious state; SVM, support vector machine; VS, vegetative state.

We first used the conventional approach (Figure 2A). Results indicated that microstates A, B, C, D, and E were detected in the control, EMCS, and MCS groups. The VS/UWS group displayed only microstates A, B, C, and E, while microstate D was not observed. Notably, there was a statistically significant increase in the GEV of microstate E in the VS/UWS group, relative to both the control and EMCS groups (Figure S1A). However, attempting to demonstrate inter‐group differences utilizing intra‐group templates proved inadequate.

In the second method, we employed an individual clustering approach (Figure 2A). We analyzed the proportion and GEV of each microstate across groups (Figure S1B). Each patient had a unique set of microstates and did not necessarily exhibit all five microstates. Moreover, the proportion and GEV of microstate E showed an increasing trend with decreasing consciousness levels, while the proportion and GEV of microstate D showed the opposite trend (Figure S1C,D). However, clustering individual topographic maps first can lead to misclassification of certain maps due to the consideration of the maximum GEV feature.

Thus, we used the third method of optimized topographic analysis (Figure 2A). As the templates of healthy controls consisted of microstates A–E, each patient contained all five microstates. The findings of this study indicate that the GEV of microstates A and E were significantly elevated in the group of patients diagnosed with VS/UWS as compared to both the healthy control and the EMCS groups. Conversely, the GEV of class D in the VS/UWS group was significantly lower than that of the healthy control group, as illustrated in Figure 2B.

The SVM regression classification of the VS/UWS group based on the GEV values of microstates E and microstates A, D, and E together calculated by the third optimized method yielded unsatisfactory results, with very low sensitivity (Figure 2C,D). Thus, the discriminatory power of the GEV in identifying VS/UWS patients was comparatively low. Please refer to Table S1 for the specific parameters of the SVM classifiers.

### 3.4 Duration and occurrence were also important markers for measuring the level of consciousness

**FIGURE 3:**
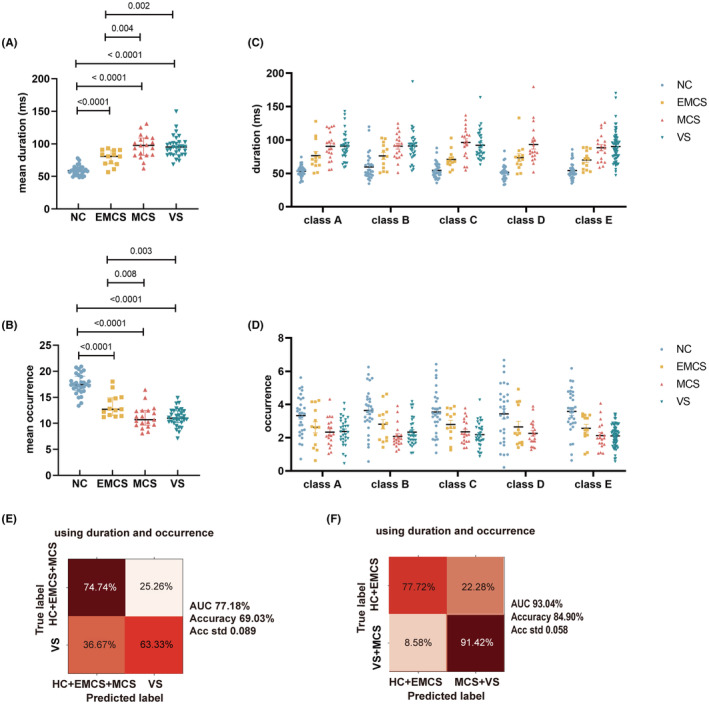
Duration and occurrence calculated by the EEGLAB plugin were also important markers for measuring the level of consciousness. (A) The mean duration of each group. (B) The duration distribution of each microstate among groups. (C) Comparison of mean occurrence among groups. (D) The occurrence of each microstate was compared among different groups. E. SVM classifier achieved 63.33% sensitivity and 74.74% specificity for diagnosing VS by duration and occurrence (AUC = 77.18%, accuracy = 69.03%). (F) SVM classifier achieved 91.42% sensitivity and 77.72% specificity for diagnosing MCS or VS/UWS by duration and occurrence (AUC = 93.04%, accuracy = 84.90%). The comparison among groups was performed by one‐way ANOVA and Bonferroni multiple tests in (A–D). AUC, area under the curve; EMCS, exit from minimal consciousness state; GEV, global explained variance; HC, healthy controls; MCS, minimally conscious state; SVM, support vector machine; VS, vegetative state.

As shown in Figure 3, the microstate parameters calculated by the EEGLAB plugin exhibited a linear path to unconsciousness. Notably, with the decline in consciousness level, the mean duration showed an increasing trend and the mean occurrence showed a decreasing trend (Figure 3A,B), regardless of the microstate category (Figure 3C,D; Tables S2 and S3). Microstates within the same group exhibited similar durations and occurrences (Figure 3C,D; Tables S2 and S3). However, the MCS and VS/UWS groups displayed similar mean durations and occurrences (Figure 3A,B), as they both exhibited very poor levels of consciousness.

The accuracy of the classifier to identify VS/UWS using mean duration and occurrence calculated by the EEGLAB plugin was 69.03%, with 63.33% sensitivity and 74.74% specificity (Figure 3E). In contrast, the accuracy to identify MCS or VS/UWS was 84.90%, with 91.42% sensitivity and 77.72% specificity (Figure 3F). The parameters of the SVM classifier are presented in Table S4. Thus, this approach exhibited limited efficacy in identifying patients with VS/UWS, probably due to the comparable mean duration and occurrence between MCS and VS/UWS.

Additionally, we compared the results of duration and occurrence using the conventional and optimized topographic analysis by Cartool software, and the comparison of inter‐group differences yielded results similar to those obtained using the EEGLAB plugin (Figure S2).

### 3.5 The GEV of microstate E played an important role in assessing the prognosis of patients with pDoC

The prognosis of patients with MCS and VS/UWS has great impacts on the whole family and society. Hence, our study centered on the prognostication of patients with MCS and VS/UWS and explored the prognostic impact of microstate parameters. Following 6 months of observation, it was determined that 7 patients diagnosed with MCS and 10 patients diagnosed with VS/UWS exhibited favorable prognoses, whereas 12 patients with MCS and 11 patients with VS/UWS demonstrated unfavorable prognoses. Table S5 presents the patient's particulars. There were no significant differences in age, sex, and months after injury between groups.

**FIGURE 4:**
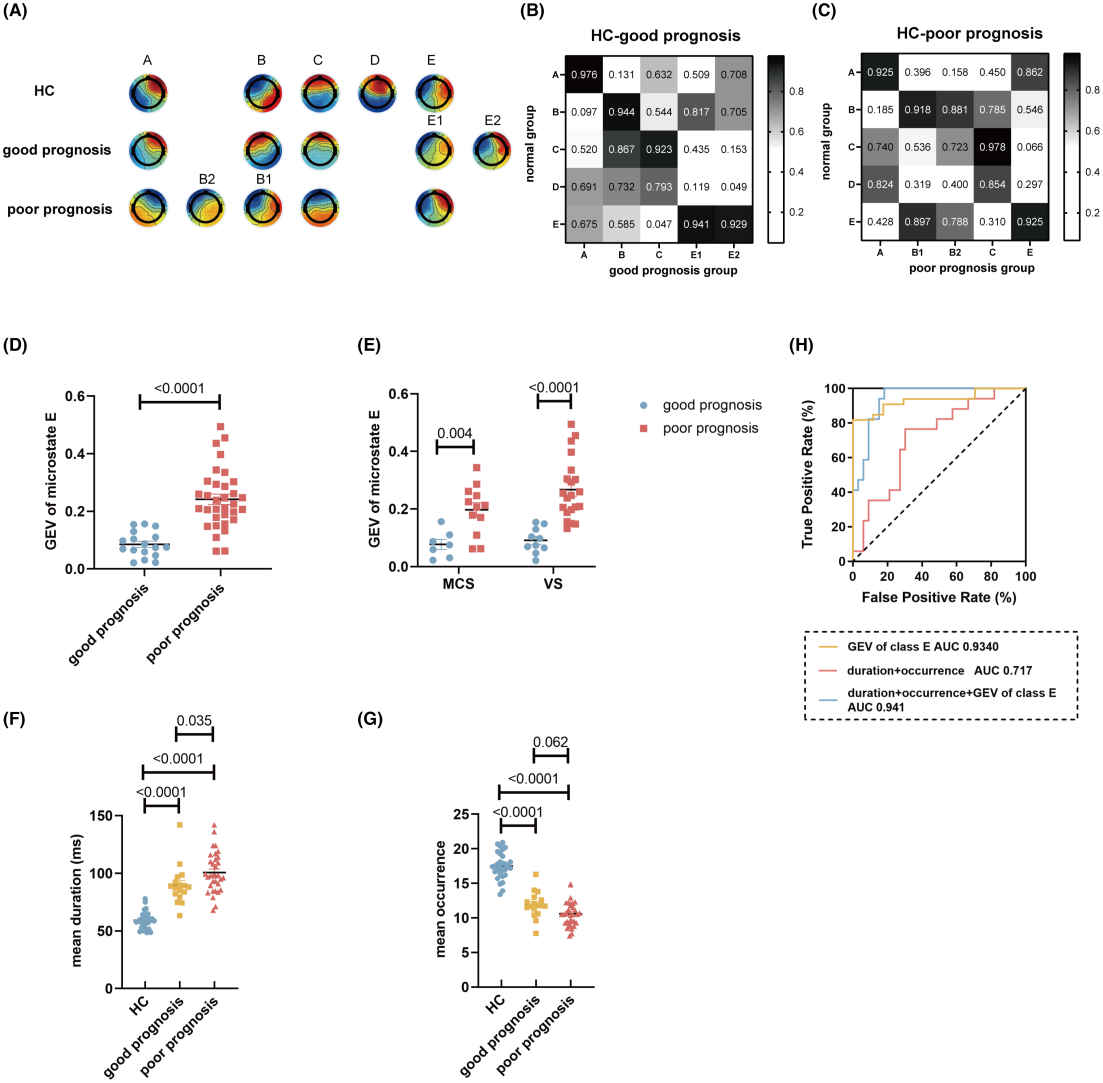
The GEV of microstate E calculated by the optimized topographic analysis was closely associated with the prognosis of pDoC. (A) Comparison of topographic maps of good and poor prognosis groups with five standard topographic maps of the control group. (B) Spatial correlation coefficient between the five microstates of the control group with five microstates of the good prognosis group. (C) Spatial correlation coefficient between the five microstates of the control group with five microstates in the poor prognosis group. (D) GEV distribution of microstate E in the good prognosis and poor prognosis groups. (E) GEV distribution of class E for MCS and VS groups. (F) Mean duration distribution of groups. (G) Mean occurrence distribution of groups. (H) ROC curve made using GEV of microstate E, duration, and occurrence. In (F) and (G), comparisons among groups were conducted using one‐way ANOVA and Bonferroni multiple tests. Independent sample *t*‐test was conducted for group comparisons in (D) and (E). AUC, area under the curve; GEV, global explained variance; HC, healthy controls; MCS, minimally conscious state; ROC, receiver operating characteristic curve; VS, vegetative state.

Using the topographic images of the control group as templates, we assigned the topographic images of the good prognosis group with microstates A, B, C, E1, and E2, and we assigned the topographic images of the poor prognosis group with microstates A, B1, B2, C, and E. Neither image of the two groups was assigned to microstate D (Figure 4A–C).

We conducted additional validation to determine the efficacy of the optimized topographic analysis in prognosticating patient outcomes. The GEV of microstate E exhibited a statistically significant increase in the poor prognosis group as compared to the good prognosis group (Figure 4D), both in the MCS and VS/UWS groups (Figure 4E). However, no significant variations were observed in the GEV of microstate E between the two groups when employing the conventional approach (Figure S3A,B). The mean duration and occurrence were calculated by the EEGLAB plugin. The mean duration of the good prognosis group was found to be significantly lower than that of the poor prognosis group, yet still higher than that of the control group (Figure 4F), while the mean occurrence exhibited an opposite outcome to the mean duration (Figure 4G). Finally, the classification performance was evaluated by utilizing these microstate parameters through the ROC curve analysis. The utilization of GEV of microstate E alone resulted in a remarkable AUC of 0.934, indicating a highly effective discrimination performance. Using duration and occurrence achieved an AUC of 0.717. The integration of GEV of microstate E, duration, and occurrence also yielded a remarkable AUC of 0.941 (Figure 4H). Thus, we proved the optimal efficiency of the GEV of microstate E in predicting the prognosis of pDOC patients.

To further confirm the prognostic significance of the GEV of microstate E computed using optimized topographic analysis, we conducted a validation study on another independent dataset. The new validation set consisted of 14 MCS patients and 16 VS/UWS patients. Among the individuals in the MCS group, 5 had a good prognosis, while the VS group had 6 individuals with a good prognosis. The detailed data of patients were provided in Table S6. The age, sex, and months after injury were similar between groups.

**FIGURE 5:**
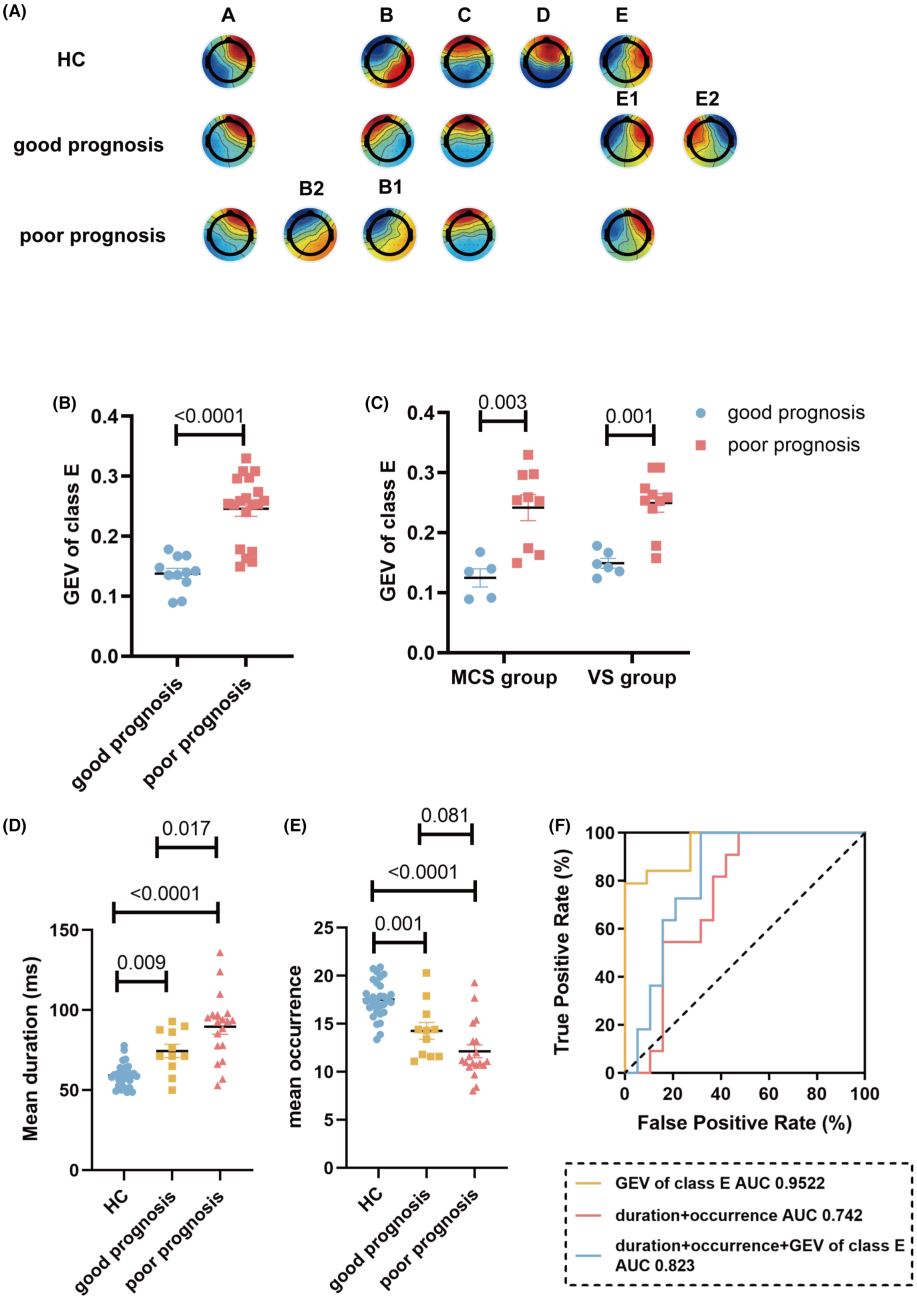
The results of the validation set using GEV of microstate E calculated by the optimized topographic analysis to predict the prognosis of pDoC. (A) The topographic maps of good and poor prognosis groups (B) GEV distribution of microstate E in the good prognosis and poor prognosis groups. (C) GEV distribution of class E for MCS and VS groups. (D) Mean duration distribution of groups. (E) Mean occurrence distribution of groups. (F) ROC curve made using GEV of microstate E, duration, and occurrence. In (D) and (E), comparisons among groups were conducted using one‐way ANOVA and Bonferroni multiple tests. Independent sample *t*‐test was conducted for group comparisons in (B) and (C). AUC, area under the curve; GEV, global explained variance; HC, healthy controls; MCS, minimally conscious state; ROC, receiver operating characteristic curve; VS, vegetative state.

The results of the validation set were consistent with the previous findings. Both two groups had microstates A, B, C, and E (Figure 5A). The optimized topographic analysis revealed a statistically significant decrease in the GEV of microstate E in the good prognosis group as compared to the poor prognosis group, in both the MCS and VS/UWS groups (Figure 5B,C). Meanwhile, the mean duration calculated by the EEGLAB plugin exhibited a significant decrease in the good prognosis group relative to the poor prognosis group, yet a notable increase in comparison to the control group (Figure 5D), while the mean occurrence exhibited an opposite outcome to the mean duration (Figure 5E). The analysis of the ROC curve indicated that the AUC for the utilization of the GEV distribution of microstate E was 0.9522; the utilization of duration and occurrence in ROC curve analysis resulted in an AUC value of 0.742; and incorporating both the GEV of microstate E and the duration and occurrence in ROC curve analysis yielded an AUC value of 0.823. These further verified the optimal efficiency of the GEV of microstate E in predicting the prognosis of pDOC patients (Figure 5F).

Finally, SVM was conducted by using GEV of class E, duration, occurrence, and their combination to assess the values of these parameters for predicting prognosis. The performance of prognosis prediction based on the GEV values of microstate E was pretty good, with an accuracy of 82.65%, 77.26% sensitivity, and 87.41% specificity (AUC = 93.02%, Figure S4A). Using the duration and occurrence, the accuracy of the classifier to identify a good prognosis was 65.74%, with 43.72% sensitivity and 78.29% specificity (AUC = 60.57%, Figure S4B). Their combination achieved an accuracy of the classifier in identifying a good prognosis of 79.83%, with 67.84% sensitivity and 88.61% specificity (AUC = 90.14%, Figure S4C). The parameters of this SVM classifier are presented in Table S7. These also verified the optimal efficiency of the GEV of microstate E in predicting the prognosis of pDOC patients.

### 3.6 The sLORETA analysis of each microstate

The present study has identified the left temporal lobe as a significant source of microstate A (Figure S5A), and the generators of microstate B were predominantly localized within the right temporal lobe (Figure S5B). Microstate C showed a high correlation with the frontal gyrus and parietal brain regions (Figure S5C), and the principal sources of microstate D were situated within the right superior and inferior parietal lobules (Figure S5D). The generators of Microstate E were predominantly situated within the medial frontal gyrus, middle frontal gyrus, and superior frontal gyrus (Figure S5E). The findings of sLORETA aligned with prior scholarly works,^8^ offering an investigation and verification of spatial activation patterns across various microstates. We presented the details of the sLORETA analysis for each microstate in Table S8.

## 4 DISCUSSION

We introduced three different approaches for estimating topographic differences among groups and identified the optimal method. Using this optimal method, it was observed that the GEV distribution of microstate E exhibited a robust prognostic capability for patients, albeit with limited discriminatory power between VS and MCS patients.

Two topographic maps of microstate E were identified in the VS/UWS group. Microstate E, rarely reported, was first discovered in semantic dementia patients by Professor Grieder.^23^ In our result, microstate E was characterized by the activity of brain regions that were typically associated with the frontal gyrus, consistent with the previous study.^8^ Microstate E is a fundamental component of the default mode network, which is known to be involved in theory of mind and mental simulations.^24^ Therefore, microstate E may have some clinical significance for assessing consciousness or predicting prognosis.

GEV is a statistical measure used to quantify the proportion of total signal variance that can be explained by microstates. The use of GEV enables us to better understand the explanatory power and relative contribution of microstates to variations in the EEG signal.^5^ This helps us determine which microstates provide more effective explanations and have a greater impact on the overall variability of the EEG signal.^25^ D'Croz‐Baron and colleagues reported a greater GEV of microstate E in the group diagnosed with ASD.^26^ We also found that the GEV of microstate E was higher in the MCS and VS groups, which suggested that microstate E was dominant for identifying unconsciousness. However, we were unable to identify a statistically significant disparity in the GEV of microstate E between the MCS and VS cohorts.

The elevated GEV of microstate E exhibited a robust association with an unfavorable prognosis among patients diagnosed with either MCS or VS. A high GEV value of Microstate E suggested that this microstate had a greater ability to explain the entire EEG signal. This implied that the energy levels of microstate E were relatively higher compared to other microstate categories. Studies by Pipinis et al.^27^ and Tarailis et al.^28^ have reported a negative correlation between microstate E and somatic awareness. Moreover, these findings have been strengthened by Tomescu et al.,^29^ who also found a negative association between microstate E and somatic awareness ratings. Therefore, higher GEV of microstate E may indicate a poorer ability to perceive the external environment, which may ultimately be associated with poor prognosis outcomes. Therefore, there was a significant difference in the GEV of microstate E between the two groups of VS patients with good and poor prognoses.

Compared to the control group, EMCS, MCS, and VS/UWS groups had significantly longer mean durations but significantly lower occurrences. The results showed that the more severe the impaired consciousness, the longer the mean duration, which may involve the interruption of information processing pathways. Sample evidence has shown that patients with MCS have longer durations than those with VS/UWS.^13^ Moreover, shorter duration and more occurrence predicted a better prognosis, suggesting that the closer the microstate parameters to normal, the better the prognosis.^9^, ^12^, ^23^

The general limitation of the new methods was that the topographic parameters did not seem to add a significant performance boost to the conventional parameters in classification for pDoC. Possible reasons for the suboptimal diagnostic efficacy of the method could be attributed to its disease specificity. In other words, although it did not perform well in accurately diagnosing the pDoC patients in question, it may exhibit relatively better diagnostic characteristics for other diseases. In addition, we are currently unable to account for the reason why the Cartool‐computed product of duration and occurrence does not equal 1.

## 5 CONCLUSION

Overall, this is the first study to propose an improved microstate method for calculating the GEV of each microstate. It provides a standardized description that offers novel avenues for the analysis of microstates. Furthermore, the higher GEV of microstate E could be used as useful markers for predicting poor prognosis of DoC, which could not be achieved by conventional methods.

## AUTHOR CONTRIBUTIONS

**Yi Ling:** Conceptualization, Methodology, Software, Formal analysis, Data curation, Writing—original draft, and Visualization. **Xinrui Wen:** Conceptualization and Writing—review and editing. **Jianghui Tang:** Conceptualization and Methodology. **Zhengde Tao:** Writing—review and editing. **Liping Sun:** Writing—review and editing. **Hailiang Xin:** Resources and Project administration. **Benyan Luo:** Conceptualization, Writing—original draft, Supervision, and Project administration.

## FUNDING INFORMATION

This work was supported by the China Brain Project 2021ZD0200404 and the National Natural Science Foundation of China (Nos. U22A20293 and 82071173).

## CONFLICT OF INTEREST STATEMENT

The authors declare no conflicts of interest.

## Supporting information


Data S1:

Data S2:

## Data Availability

Data can be downloaded from FigShare and the download address is as follows: https://doi.org/10.6084/m9.figshare.23552964.v1. Other detailed information regarding the methods is provided in the supplementary methods.
