# Supplementary material for: Effect of topographic comparison of electroencephalographic microstates on the diagnosis and prognosis prediction of patients with prolonged disorders of consciousness
Source: CNS Neurosci Ther. 2023 Sep 7;30(3):e14421. doi: 10.1111/cns.14421 (PMC10915977; doi:10.1111/cns.14421)
Supplement: Supplementary file 1 — Data S1: [file CNS-30-e14421-s001.docx]

Fig. S1


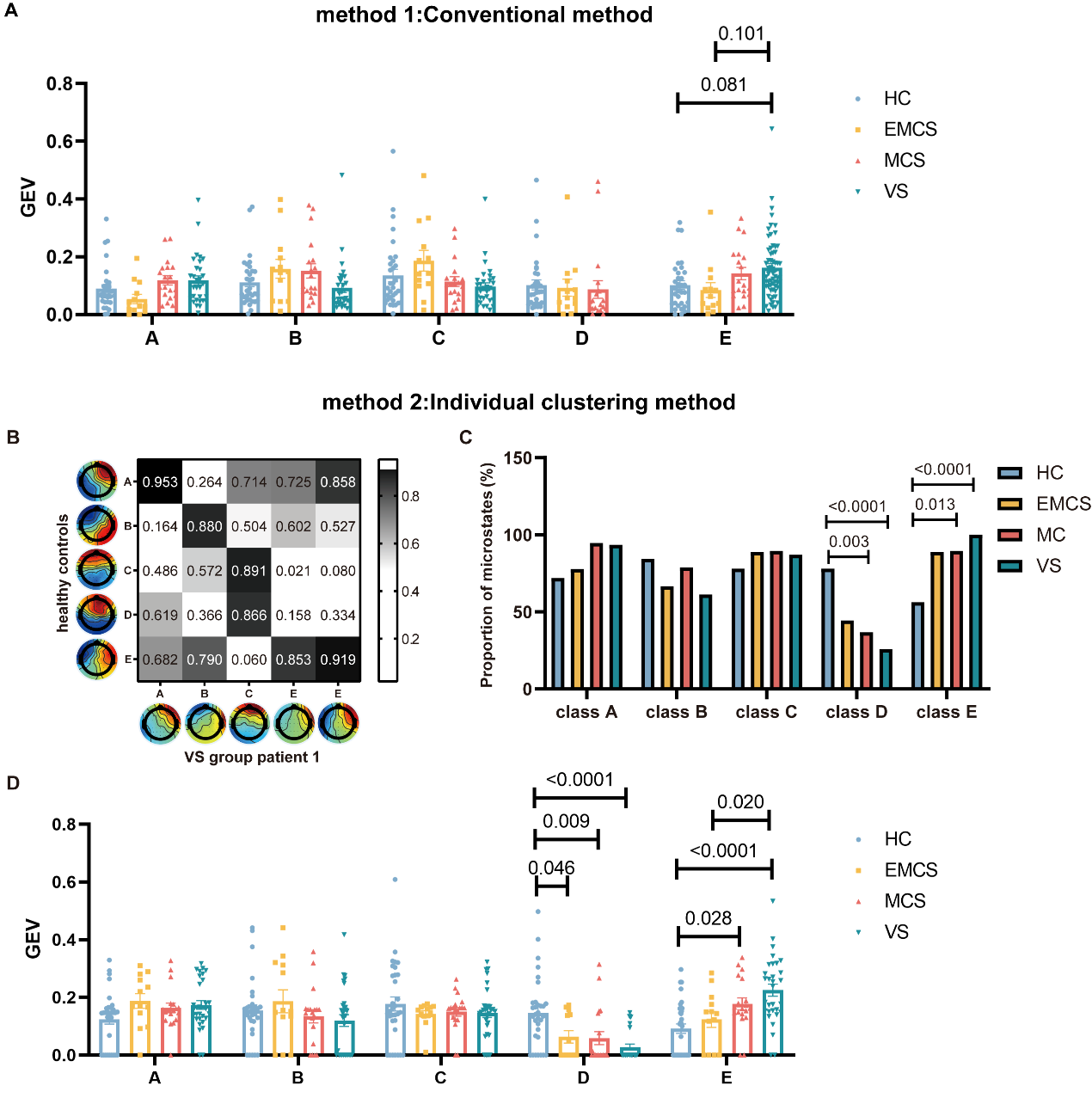


Fig. S1 The conventional method and individual clustering method. A. GEV the distribution of each microstate across groups using the conventional method. B. Aggregate maps for each individual were assigned to aggregate maps of the control group based on the maximal absolute value of Pearson correlation. C. According to the five individual microstates of each group corresponding to the five standard microstates of the control group, the proportion of five microstates in each group was determined. D. Distribution of GEV of each microstate across groups. The comparison among groups was performed by one-way ANOVA and Bonferroni multiple tests in panels A and D. The comparison among groups was conducted by chi-square test in panels C. HC, healthy controls; EMCS, exit from minimal consciousness state; MCS, minimally conscious state; VS, vegetative state; GEV, global explained variance.


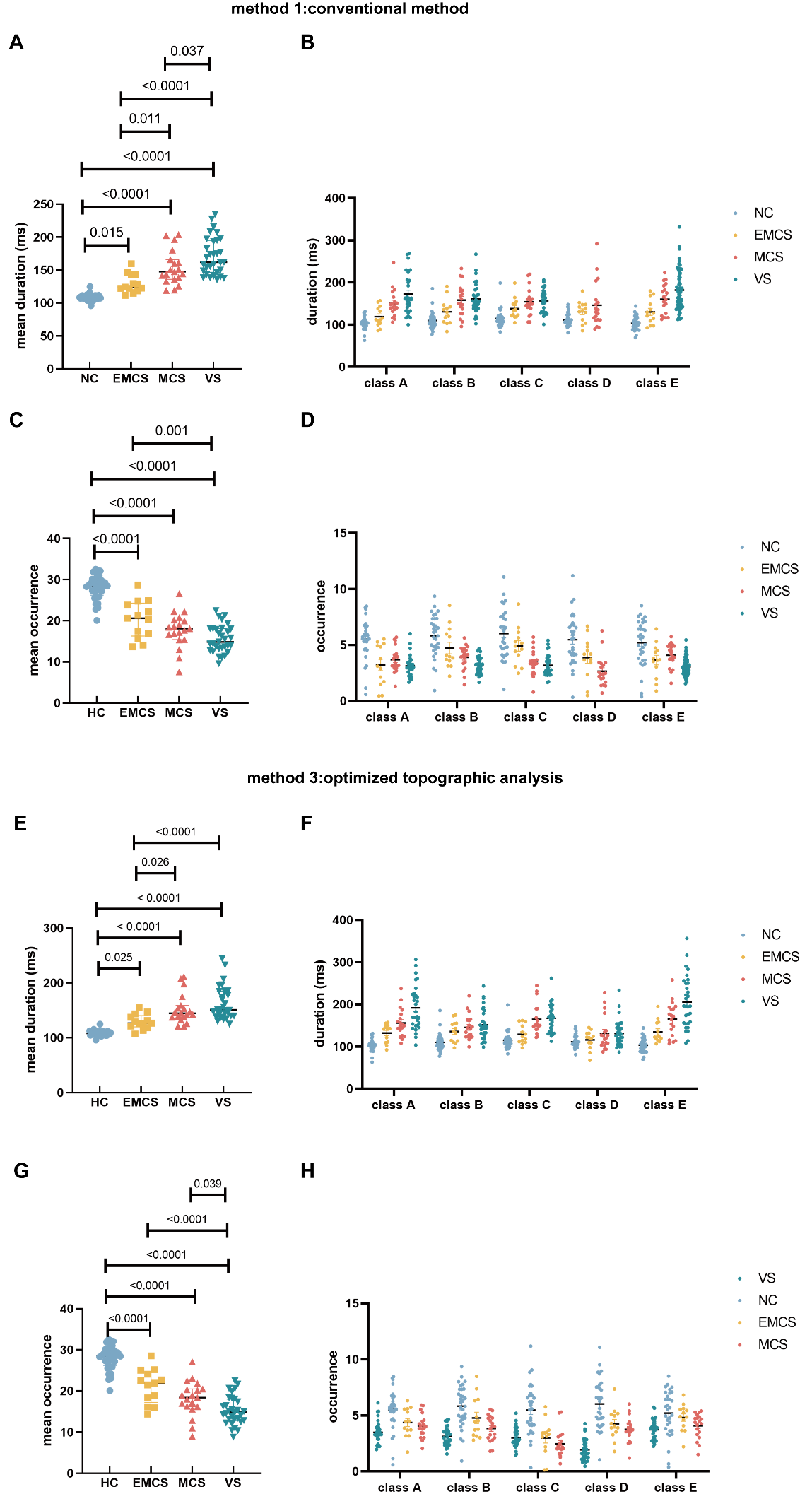


Fig. S2

Fig. S2 Microstate parameters calculated by conventional method and optimized topographic analysis using Cartool software. A-D. Duration and occurrence conducted by conventional method. E-H. Duration and occurrence conducted by optimized topographic analysis. The comparison between groups was performed by one-way ANOVA and Bonferroni multiple tests in A, C, E and G. HC, healthy controls; EMCS, exit from minimal consciousness state; MCS, minimally conscious state; VS, vegetative state.

Fig. S3


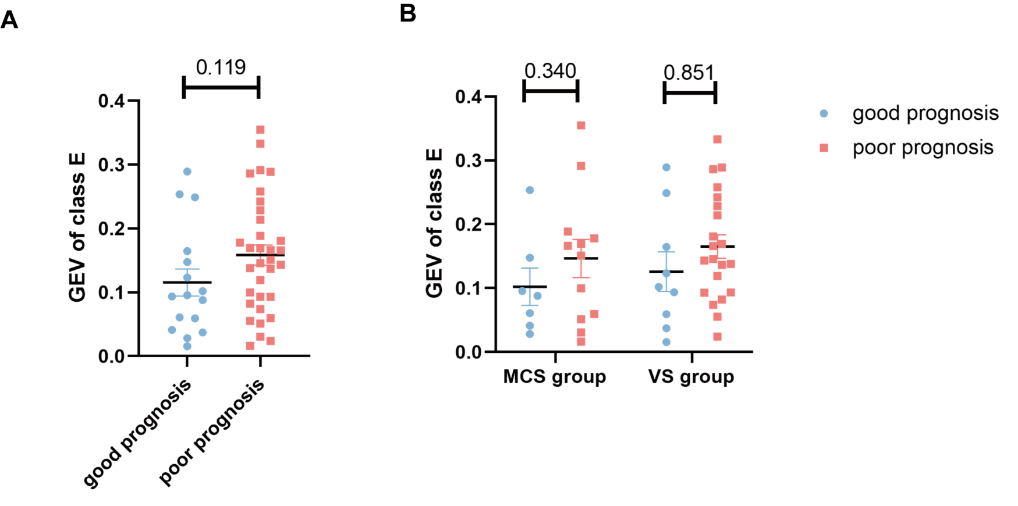


Fig. S3 The role of GEV of class E calculated by conventional method for predicting prognosis. A. The GEV of class E distribution between good prognosis and poor prognosis. B. The distribution of GEV of microstate E for MCS and VS groups. The comparison between groups was performed by independent sample T test. GEV, global explained variance MCS, minimally conscious state; VS, vegetative state.


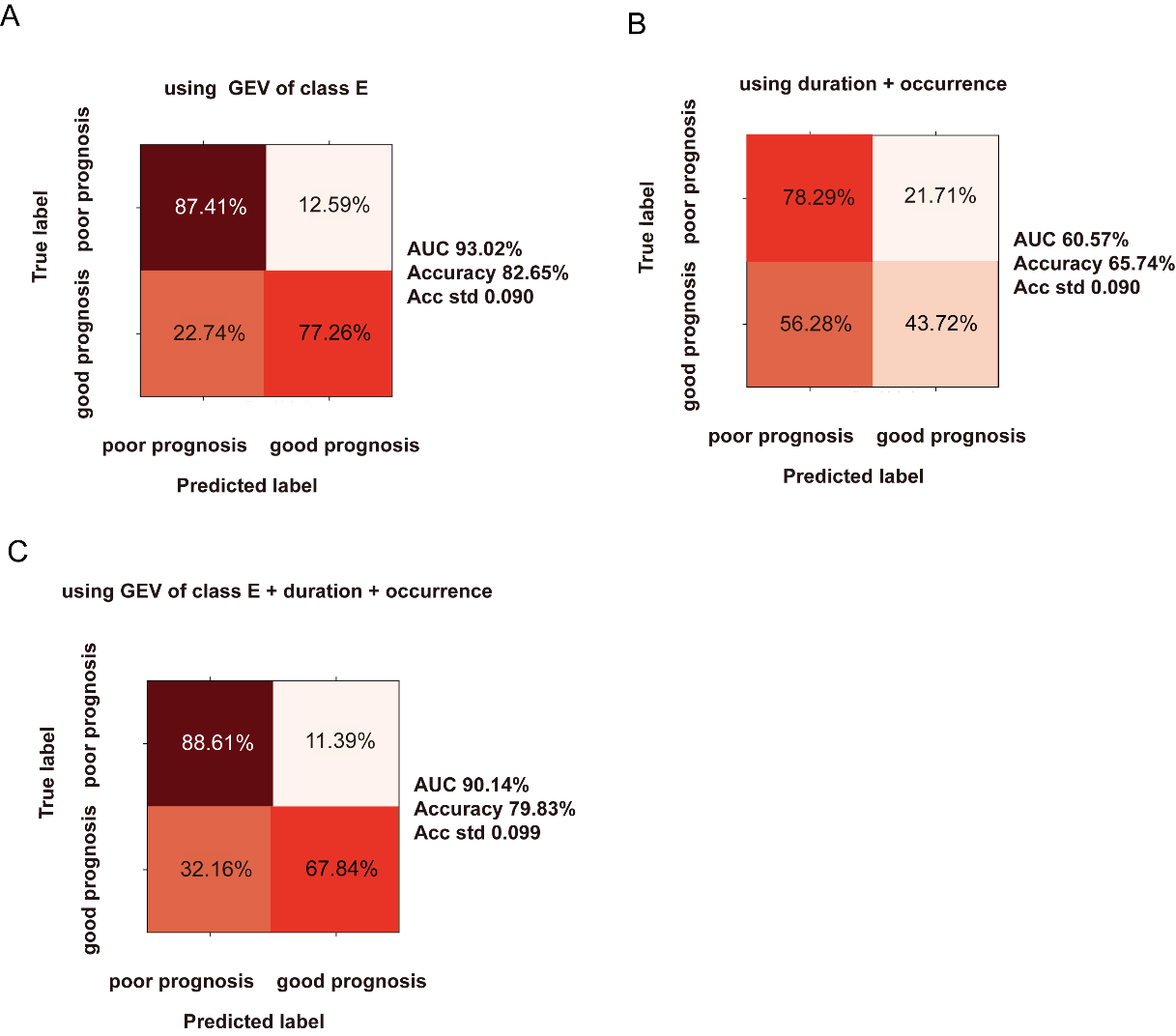


Fig. S4

Fig. S4 The role of GEV of class E, duration and occurrence for assessing the prognosis of patients. A. GEV of microstate E was used to test the prediction efficiency of the SVM model for identifying good prognosis group. Results of the confusion matrix showed 77.26% sensitivity and 87.41% specificity for good prognosis diagnosis. B. Duration and occurrence showed 43.72% sensitivity and 78.29% specificity for good prognosis diagnosis. C. The SVM classifier trained using the GEV of microstate E, duration and occurrence showed 67.84% sensitivity and 88.61% specificity for good prognosis diagnosis (AUC = 90.14%, accuracy = 79.83%).


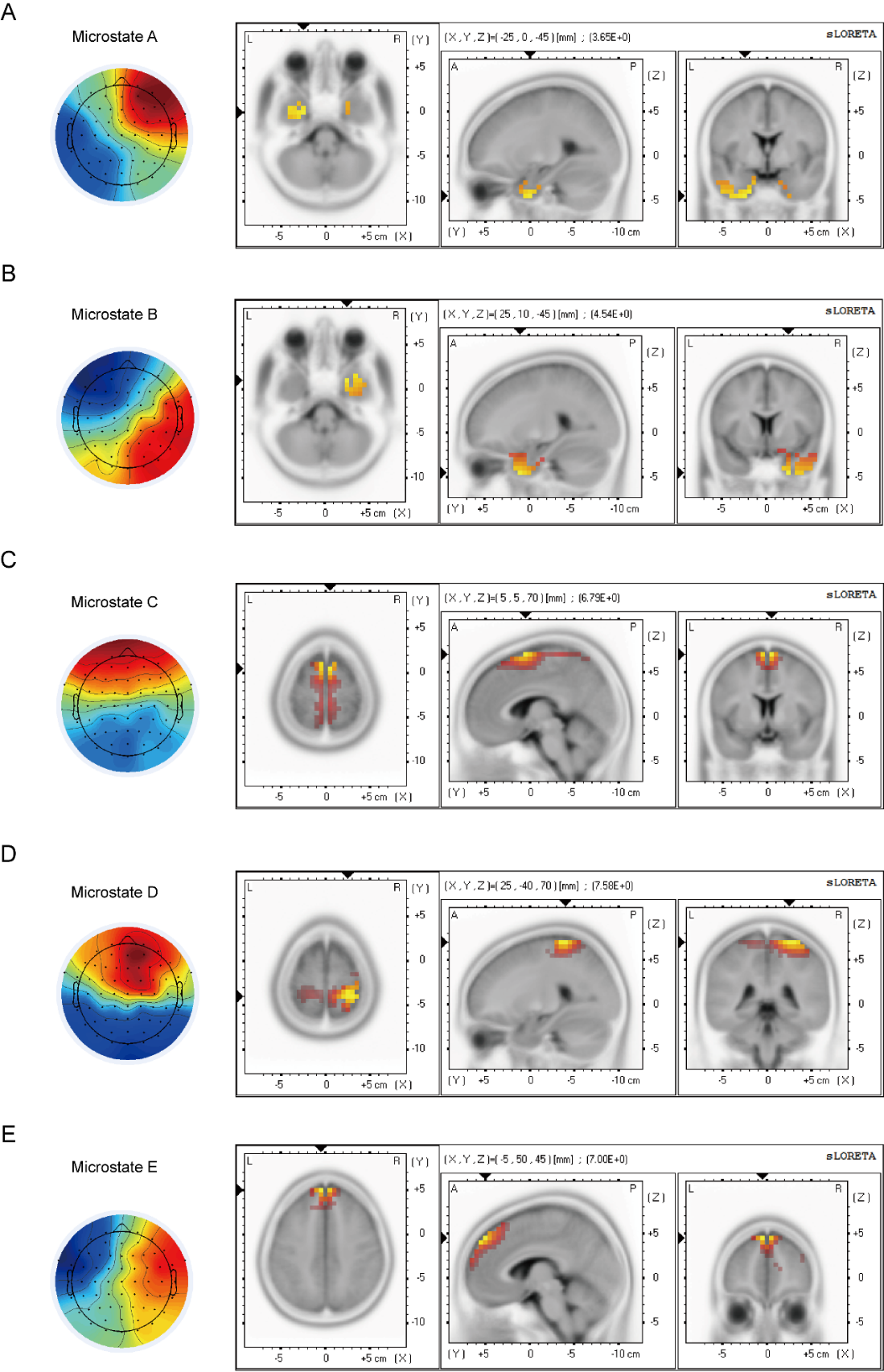


Fig. S5

Fig. S5 Activation of each microstate conducted by sLORETA in the healthy control group. A. The source localization results of microstate A (z score>3). B. The source localization results of microstate B (z score>3). C. The source localization results of microstate C (z score>3). D. The source localization results of microstate D (z score>3). E. The source localization results of microstate E (z score>3).

**Table S1. For disease classification using GEV**

We used 5-fold cross-validation and determined the optimal model parameters

| Data | GEV of E | GEV of A+D+E |
| --- | --- | --- |
| Label (1) | VS | VS |
| the optimal model parameters | Kernel=linear | Kernel=RBF |
|  | C=5 | C=6 |
|  | Gamma=0.00001 | Gamma=1.0 |

Abbreviations: SVM, support vector machine; GEV, global explained variance; RBF, radial basis function.

**Table S2 Microstate duration for microstate class A to E**

|  | **HC** | **EMCS** | **MCS** | **VS** | **Adjusted *P* Value** | | | | | |
| --- | --- | --- | --- | --- | --- | --- | --- | --- | --- | --- |
|  |  |  |  |  | **HC-**  **EMCS** | **HC-**  **MCS** | **HC-**  **VS** | **EMCS-**  **MCS** | **EMCS-**  **VS** | **MCS-**  **VS** |
| **A** | 53.2 | 76.4 | 90.5 | 90.8 | **0.002** | **<0.0001** | **<0.0001** | 0.228 | 0.127 | 1.000 |
| **B** | 59.5 | 76.0 | 90.5 | 90.9 | 0.143 | **<0.0001** | **<0.0001** | 0.395 | 0.241 | 1.000 |
| **C** | 54.4 | 70.8 | 96.2 | 92.0 | 0.055 | **<0.0001** | **<0.0001** | **0.002** | **0.005** | 1.000 |
| **D** | 51.6 | 73.5 | 93.2 | 89.7 | **0.017** | **<0.0001** | **<0.0001** | 0.077 | 0.151 | 1.000 |
| **E** | 54.2 | 70.0 | 88.3 | 90.6 | 0.090 | **<0.0001** | **<0.0001** | 0.054 | **0.009** | 1.000 |

Multiple comparisons were conducted using one-way ANOVA, and P-values were adjusted using the Bonferroni method. Bold text indicates a statistically significant difference. Abbreviations: HC, healthy controls; EMCS, exit from minimal consciousness state; MCS, minimally conscious state; VS, vegetative state.

**Table S3 Microstate occurrence for microstate class A to E**

|  | **HC** | **EMCS** | **MCS** | **VS** | **Adjusted *P* Value** | | | | | |
| --- | --- | --- | --- | --- | --- | --- | --- | --- | --- | --- |
|  |  |  |  |  | **HC-**  **EMCS** | **HC-**  **MCS** | **HC-**  **VS** | **EMCS-**  **MCS** | **EMCS-**  **VS** | **MCS-**  **VS** |
| **A** | 3.3 | 2.6 | 2.3 | 2.4 | 0.237 | **0.007** | **0.002** | 1.000 | 1.000 | 0.833 |
| **B** | 3.6 | 2.8 | 2.1 | 2.3 | 0.114 | **<0.0001** | **<0.0001** | 0.307 | 1.000 | 1.000 |
| **C** | 3.5 | 2.8 | 2.4 | 2.2 | 0.159 | **0.001** | **<0.0001** | 1.000 | 0.407 | 1.000 |
| **D** | 3.4 | 2.7 | 2.3 | 2.1 | 0.321 | **0.008** | **<0.001** | 1.000 | 1.000 | 1.000 |
| **E** | 3.6 | 2.6 | 2.1 | 2.1 | **0.012** | **<0.0001** | **<0.0001** | 1.000 | 0.989 | 1.000 |

Multiple comparisons were conducted using one-way ANOVA, and P-values were adjusted using the Bonferroni method. Bold text indicates a statistically significant difference. Abbreviations: HC, healthy controls; EMCS, exit from minimal consciousness state; MCS, minimally conscious state; VS, vegetative state.

**Table S4. For disease classification using traditional parameters**

We used 5-fold cross-validation and determined the optimal model parameters

| Data | duration + occurrence | duration + occurrence |
| --- | --- | --- |
| Label (1) | VS | VS+MCS |
| the optimal model parameters | Kernel=linear | Kernel=linear |
|  | C=1 | C=1 |
|  | Gamma=0.00001 | Gamma=0.00001 |

Abbreviations: MCS, minimally conscious state; VS, vegetative state.

**Table S5**

**Clinical data of improved and unimproved groups**

| **Characteristics** | **improved (n = 17)** | **unimproved (n = 33)** | ***P* - value** |
| --- | --- | --- | --- |
| Age | 67.29(7.39) | 66.82(5.09) | 0.790 |
| Sex (male %) | 52.9 | 45.5 | 0.616 |
| GOS^*^ | 4(4,5) | 3(2,3) | <0.0001 |
| Months after injury | 8.27(1.22) | 7.78(1.31) | 0.210 |

One way ANOVA for age and Months after injury analysis

Chi-Square for sex analysis

Kruskal-Wallis Tests for GOS analysis

^*^ *P* < 0.05

Abbreviation: GOS, Glasgow Outcome Scale.

**Table S6**

**Clinical data of validation**

| **Characteristics** | **improved (n = 11)** | **unimproved (n = 19)** | ***P* - value** |
| --- | --- | --- | --- |
| Age | 68.00(3.46) | 67.21(3.49) | 0.554 |
| Sex (male %) | 63.6 | 42.1 | 0.256 |
| GOS^*^ | 4(4,5) | 2(1,2) | <0.0001 |
| Months after injury | 7.96(0.981) | 8.19(0.684) | 0.454 |

One way ANOVA for age and Months after injury analysis

Chi-Square for sex analysis

Kruskal-Wallis Tests for GOS analysis

^*^ *P* < 0.05

Abbreviation: GOS, Glasgow Outcome Scale.

**Table S7. For disease prognosis prediction using traditional parameters and GEV**

We used 5-fold cross-validation and determined the optimal model parameters

| Data | GEV of E | duration + occurrence | duration + occurrence + GEV of E |
| --- | --- | --- | --- |
| Label (1) | Good prognosis | Good prognosis | Good prognosis |
| the optimal model parameters | Kernel=RBF | Kernel=RBF | Kernel=linear |
|  | C=6 | C=6 | C=6 |
|  | Gamma=1.0 | Gamma=1.0 | Gamma=0.00001 |

Abbreviations: GEV, global explained variance; RBF, radial basis function.

**Table S8**

**Brain regions which are activated in microstates**

| **Microstate A** | **Microstate B** | **Microstate C** | **Microstate D** | **Microstate E** |
| --- | --- | --- | --- | --- |
| Left Inferior Temporal Gyrus | Right Inferior Temporal Gyrus | Precuneus | Right Superior Parietal Lobule | Medial Frontal Gyrus |
| Left Middle Temporal Gyrus | Right Middle Temporal Gyrus | Paracentral Lobule | Right Inferior Parietal Lobule | Middle Frontal Gyrus |
| Left Superior Temporal Gyrus | Right Superior Temporal Gyrus | Precentral Gyrus | Postcentral Gyrus | Superior Frontal Gyrus |
| Superior Frontal Gyrus | Inferior Frontal Gyrus | Postcentral Gyrus | Precentral Gyrus |  |
| Rectal Gyrus | Fusiform Gyrus | Medial Frontal Gyrus | Sub-Gyral |  |
|  | Rectal Gyrus | Superior Frontal Gyrus | Paracentral Lobule |  |

Abbreviations: brain regions with z-scores > 3 as activation areas for each microstate.
