# Supplementary material for: Effect of topographic comparison of electroencephalographic microstates on the diagnosis and prognosis prediction of patients with prolonged disorders of consciousness
Source: CNS Neurosci Ther. 2023 Sep 7;30(3):e14421. doi: 10.1111/cns.14421 (PMC10915977; doi:10.1111/cns.14421)
Supplement: Supplementary file 2 — Data S2: [file CNS-30-e14421-s002.docx]

**Methods and Materials**

**Recruitment process**

Four groups of participants took part in the experiment: VS/UWS (n = 31), MCS (n = 19), exit from minimal consciousness state (EMCS) (n = 13), and healthy controls (n = 32). When patients with MCS regain their ability to communicate functionally and manipulate objects, they should be reclassified as EMCS (Di et al., 2017, Fitzpatrick-DeSalme et al., 2022). The validation set, which included 14 MCS and 16 VS/UWS patients, was enrolled from another study of pDoC (Xu et al., 2021). Flowchart of this experiment is as follows: First, we collected EEG data within three days of admission. The Coma Recovery Scale-Revised (CRS-R) was performed five times within 10 days when the patients were awake by two trained neurologists after admission, and the highest score determined the classification of pDoC. A follow-up assessment using the Glasgow Outcome Scale (GOS) was conducted by telephone with the patient’s families 6 months after the EEG collection (McMillan et al., 2016). A GOS score ≥ 4 points is considered to indicate a good prognosis, while a GOS score < 4 points is considered to indicate a poor prognosis (Hanko et al., 2021).

The GOS scoring criteria are as follows:

| Score | Description |
| --- | --- |
| 5 | a good return to normal life, albeit with mild impairment |
| 4 | mild disability but can live independently; able to work under protection |
| 3 | severe disability, stay awake; disabled, need care in daily life |
| 2 | plants survive with minimal response, such as the ability to open their eyes with sleep and wake cycles |
| 1 | Death |

**EEG recording and data processing**

All patients with pDoC and controls stayed in a dimly lit room with a quiet environment, sitting or lying. During acquisition, patients diagnosed with pDoC were advised to maintain an open-eyed state. During the process of EEG data collection, in the event that a patient was to close their eyes, a temporary pause would be initiated, followed by the application of moderate force to the patient's hand, foot, or face as a means of prompting the patient to open their eyes. It was imperative to observe a waiting period of approximately 30 seconds subsequent to the patient's eye opening in order to ensure the stabilization of EEG signals prior to the resumption of data collection. The healthy controls were requested to open eyes. The 5 min EEG data was recorded using the international 10–20 system with 64-electrodes actiCAP (Brain Products DmbH, Munich, Germany). AFz and FCz were used as the ground and reference electrodes, respectively. The initial sampling frequency was 5000 Hz, which was subsequently reduced to 250 Hz through offline downsampling. EEG data were preprocessed using MATLAB scripts derived from the EEGLAB toolbox: remove useless electrodes, filter between 0.1 and 40 Hz, split into 2-second segments; bad channels had been interpolated, using independent component analysis, remove noncerebral artifacts; re-reference artifact-free EEG data to the average. The MATLAB 2013b software (MathWorks, Inc., Natick, MA, USA) was utilized to execute all procedures.

**Calculation formulas**

$$GFP(t)=\sqrt{\frac{\sum_{i=1}^{n} \left(v_{i}(t)-\bar{v}(t)\right)^{2}}{n}}$$

Here, vi(t) refers to the voltage reading at a specific electrode i at a given time t. At time t, $\bar{v}(t)$ represents the mean voltage measurement obtained from all electrodes. The symbol "n" represents the aggregate count of electrodes employed.

$$C=\frac{\sum_{i=1}^{n} \left(u_{i}\bullet v_{i}\right)}{\sqrt{\sum_{i=1}^{n} u_{i}^{2}}\bullet\sqrt{\sum_{i=1}^{n} v_{i}^{2}}}$$

The correlation measure is comparable to the Pearson product-moment correlation coefficient which is used to assess the relationship between two topographic maps or surfaces. The summation is performed over all i electrodes.

$$GEV=\frac{\sum_{i=1}^{m} \left(GFP^{2}\cdot C^{2}\right)}{\sum_{i=1}^{m} GFP^{2}}$$

The value of m represents the total number of maps that were originally there

Duration = Sum (duration_i) / n, duration_i represents the duration of each microstate i, and n represents the total number of microstate i.

Occurrence = Sum (occurrence_i)/ T, occurrence_i represents the number of each microstate i, and T represents the total time.

**Source localization**

The present study utilized standardized low-resolution electromagnetic tomography (sLORETA) analysis to acquire current density images for each microstate and group (Laxton et al., 2010, Mobascher et al., 2009). First, sLORETA is used to generate an electrode coordinate file, which is converted into a matrix file. To analyze the brain activity, we used a cross-spectrum file that was created from an EP file containing 5 aggregate microstates of healthy controls. By utilizing a dense grid consisting of 6239 voxels, we were able to compute the electrical activity of every microstate. We then analyzed the z-scores of the cross-spectrum files and identified brain regions with z-scores > 3 as activation areas for each microstate (Custo et al., 2017).

Z =（x-μ）/σ

The variable x denotes a singular raw data point, whereas μ signifies the average value of the entire population, and σ denotes the standard deviation of the population in its entirety.

**Machine learning**

To assess whether these topographic parameters were appropriate for the classification and prognosis of pDoC, support vector machine (SVM) regression was used to predict the classification and prediction performance of pDoC with these new parameters. Classification and prediction were performed using SVM regression code in Python. The entire dataset was partitioned into training and test sets at an 8:2 ratio using a random state and five-fold cross-validation; that is, 80% of the participants were used for training and 20% for testing. We repeated the entire procedure 1000 times, and participants were randomly assigned to training and test sessions. Our null distribution was tested by randomly swapping microstate parameters across participants and repeating the prediction procedure 10,000 times. The above procedures were performed using Python 3.9.

Reference

Custo A, Van De Ville D, Wells WM, Tomescu MI, Brunet D, Michel CM. Electroencephalographic Resting-State Networks: Source Localization of Microstates. Brain Connect 2017;7(10):671-82.

Di H, He M, Zhang Y, Cheng L, Wang F, Nie Y, et al. Chinese translation of the Coma Recovery Scale-Revised. Brain Inj 2017;31(3):363-5.

Fitzpatrick-DeSalme E, Long A, Patel F, Whyte J. Behavioral Assessment of Patients With Disorders of Consciousness. J Clin Neurophysiol 2022;39(1):4-11.

Hanko M, Grendar M, Snopko P, Opsenak R, Sutovsky J, Benco M, et al. Random Forest-Based Prediction of Outcome and Mortality in Patients with Traumatic Brain Injury Undergoing Primary Decompressive Craniectomy. World Neurosurg 2021;148:e450-e8.

Laxton AW, Tang-Wai DF, McAndrews MP, Zumsteg D, Wennberg R, Keren R, et al. A phase I trial of deep brain stimulation of memory circuits in Alzheimer's disease. Annals of neurology 2010;68(4):521-34.

McMillan T, Wilson L, Ponsford J, Levin H, Teasdale G, Bond M. The Glasgow Outcome Scale - 40 years of application and refinement. Nature reviews Neurology 2016;12(8):477-85.

Mobascher A, Brinkmeyer J, Warbrick T, Musso F, Wittsack HJ, Stoermer R, et al. Fluctuations in electrodermal activity reveal variations in single trial brain responses to painful laser stimuli--a fMRI/EEG study. Neuroimage 2009;44(3):1081-92.

Xu C, Zou J, He F, Wen X, Li J, Gao J, et al. Neural Tracking of Sound Rhythms Correlates With Diagnosis, Severity, and Prognosis of Disorders of Consciousness. Front Neurosci 2021;15:646543.
